# Robotic mitral valve surgery

**DOI:** 10.3389/fcvm.2023.1239742

**Published:** 2024-03-05

**Authors:** John Massey, Kenneth Palmer, Omar Al-Rawi, Owen Chambers, Tim Ridgway, Selvaraj Shanmuganathan, Gopal Soppa, Paul Modi

**Affiliations:** Department of Cardiothoracic Surgery, The Liverpool Heart and Chest Hospital NHS Foundation Trust, Liverpool, United Kingdom

**Keywords:** mitral valve, robotic, minimally invasive, mitral valve repair, outcomes

## Abstract

Totally endoscopic robotic mitral valve repair is the least invasive surgical therapy for mitral valve disease. Robotic mitral valve surgery demonstrates faster recovery with shorter hospital stays, less morbidity, and equivalent mortality and mid-term durability compared to sternotomy. In this review, we will explore the advantages and disadvantages of robotic mitral valve surgery and consider important technical details of both operative set-up and mitral valve repair techniques. The number of robotic cardiac surgical procedures being performed globally is expected to continue to rise as experience grows with robotic techniques and increasing numbers of cardiac surgeons become proficient with this innovative technology. This will be facilitated by the introduction of newer robotic systems and increasing patient demand.

## Introduction

Mitral valve surgery is now in its one hundredth year of practice with the first successful mitral valve repair performed by Elliot Cutler at the Brigham and Women's Hospital in 1923 (1). Over this time, the practice of mitral valve surgery has undergone numerous advances, from closed commissurotomy to the complex repair techniques of the modern era. Access to the mitral valve has seen developments too, from median sternotomy and thoracotomy to minimal access techniques and totally endoscopic robotic approaches using robotic telemanipulation. There is also evolving technology that avoids the use of cardiopulmonary bypass altogether, such as transcatheter and transapical neochords, clips and valves, and future research will help identify which subgroups are best treated with these. Thus, cardiologists and surgeons now have an armamentarium of treatments available to address different aetiologies of mitral valve dysfunction in differing risk strata of patients.

### The era of mitral valve repair

A better understanding of the natural history of untreated severe MR, which eventually leads to a decline in LV function, pulmonary hypertension, and atrial fibrillation (AF) (2), as well as the development of durable repair techniques by Dr. Alain F. Carpentier has led to degenerative valvular disease becoming the predominant aetiology of those patients undergoing mitral valve repair in the Western world. Dr. Carpentier's presentation to the 1983 meeting of the American Association of Thoracic Surgery of his paper “The French Correction” set out a blueprint for surgeons to classify mitral valve disease in terms of aetiology, lesions, and dysfunction. Mitral valve reconstruction confers numerous benefits over prosthetic replacement including lower operative morbidity and mortality, and superior long-term survival. This is due to maintenance of LV geometry/function due to preservation of all chordal attachments and avoidance of prosthetic valve complications such as structural valve degeneration, haemorrhage, and thromboembolism. Reconstructive mitral surgery and minimally invasive/robotic surgery have become subspecialisms of cardiac surgery and surgeon/institution volume has been demonstrated to correlate with outcomes (3).

The classic operation for posterior mitral valve leaflet (PMVL) prolapse associated with excess tissue that Carpentier described was a *triangular* or *quadrangular* resection, with/without annular compression or plication, and/or leaflet sliding plasty. The normal systolic dimensions of the annulus were restored with a true-sized annuloplasty ring based on the surface area of the anterior leaflet. The seminal work of Drs Frater and David (4, 5) introduced the use of Gore-Tex neochords for mitral valve repair and this led to the concept of ‘respect’ rather than ‘resect’ (6). Neochordal techniques may increase the likelihood of a successful mitral valve repair and are associated with more favourable valve haemodynamics (lower transmitral gradient and larger orifice area) when compared to leaflet resection (7, 8). Accurate judgement of length against a non-prolapsing reference point is critical and one disadvantage of ePTFE (expanded polytetrafluoroethylene) is that it is slippery, and knots can slide during tying.

In 2000, Mohr devised a method of creating pre-measured loops of ePTFE, which negated the risk of inadvertently shortening the chordal length during tying. It has proved particularly useful for minimally invasive surgery and is widely used in Europe, particularly Germany (9). The largest published experience to date was from Leipzig in 2,134 consecutive patients undergoing minimally invasive mitral valve repair using loops alone (82.1%) or resection alone (17.9%) with 10-year follow-up (mean 6.1 ± 4.3 years) (10). Leaflet resection was associated with more ≥2+ MR on predischarge echocardiography (*p* = 0.003) and was a significant predictor of late mortality. Freedom from re-operation was low in both groups at all time points (1, 5 and 10 years) with no significant difference.

### Minimal access and robotic techniques

The advantages garnered by limiting surgical trauma, through minimized incision size and avoidance of rib-spreading, have resulted in increasing numbers of surgeons adopting minimally invasive cardiac surgical (MICS) techniques to benefit their patients. This has led to a significant increase in the number of MICS procedures being undertaken internationally, such that >50% of isolated mitral valve disease in Germany is now operated on using minimally invasive (predominantly non-robotic) techniques (11). Improvements in surgical techniques (e.g., Gore-Tex loops), instrumentation (shafted instruments), perfusion technology (thin-walled reinforced cannulae) and vision platforms (3D video stacks) have helped disseminate MICS techniques. Minimally invasive procedures are thus as safe, effective, and durable as conventional surgery.

However, shafted endoscopic instruments used during non-robotic ‘*mini mitral’* surgery limit surgical dexterity. As incision size decreases, three things happen:

(1) The ***fulcrum effect*** where the tool endpoints move in the opposite direction to the surgeon’s hands due to the pivot point at the chest wall, making totally endoscopic surgery more difficult to learn.
(2) Direct vision surgery becomes challenging due to the ***loss of depth perception*** from using two-dimensional video monitors which further increases operative difficulty.
(3) Control of the aortic root becomes more challenging and a transition from direct cannulation with Chitwood transthoracic clamping to endoaortic balloon occlusion becomes necessary.

**Figure 1:**
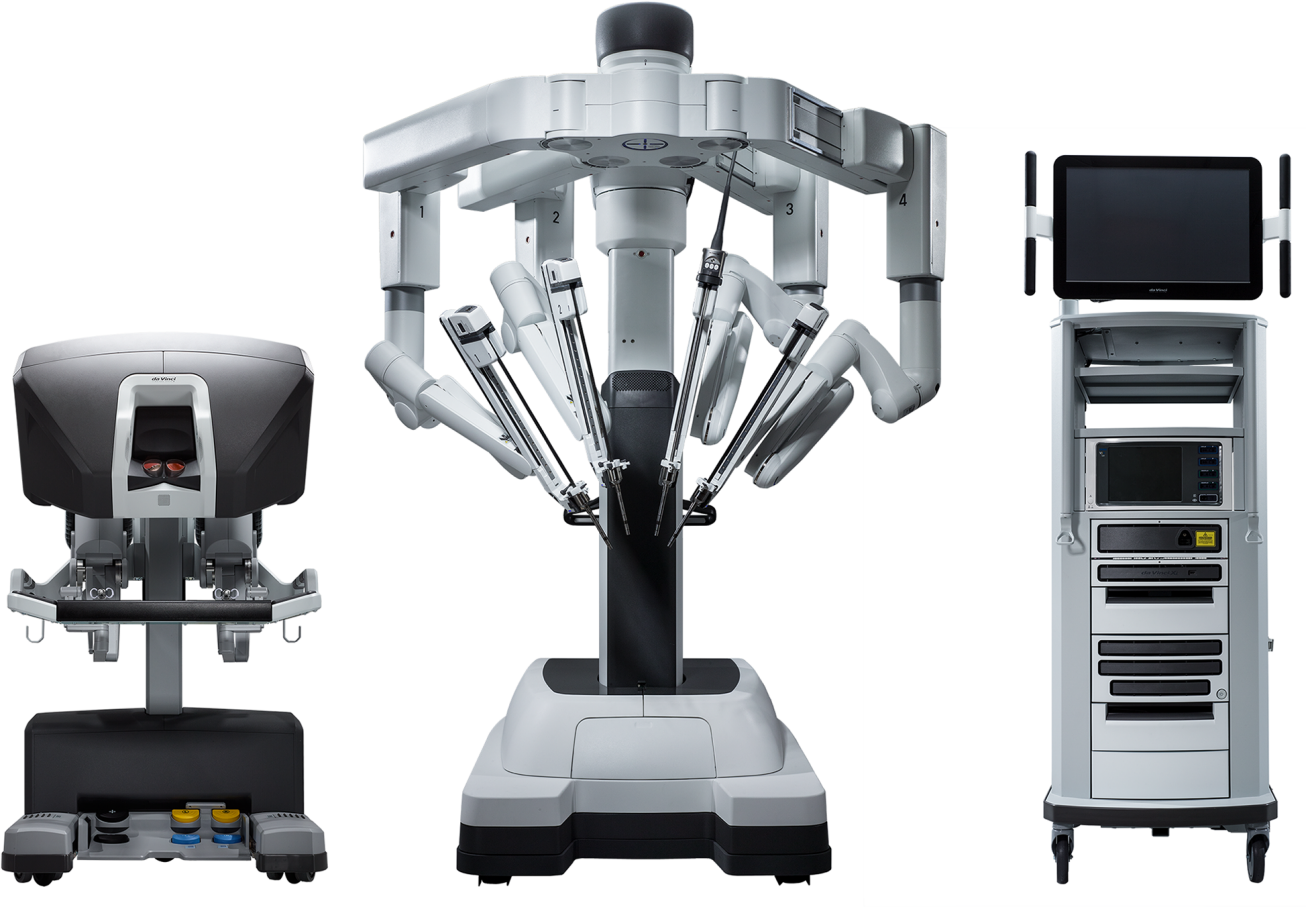
The Da Vinci Xi (reproduced with permission from intuitive surgical).

**Figure 2:**
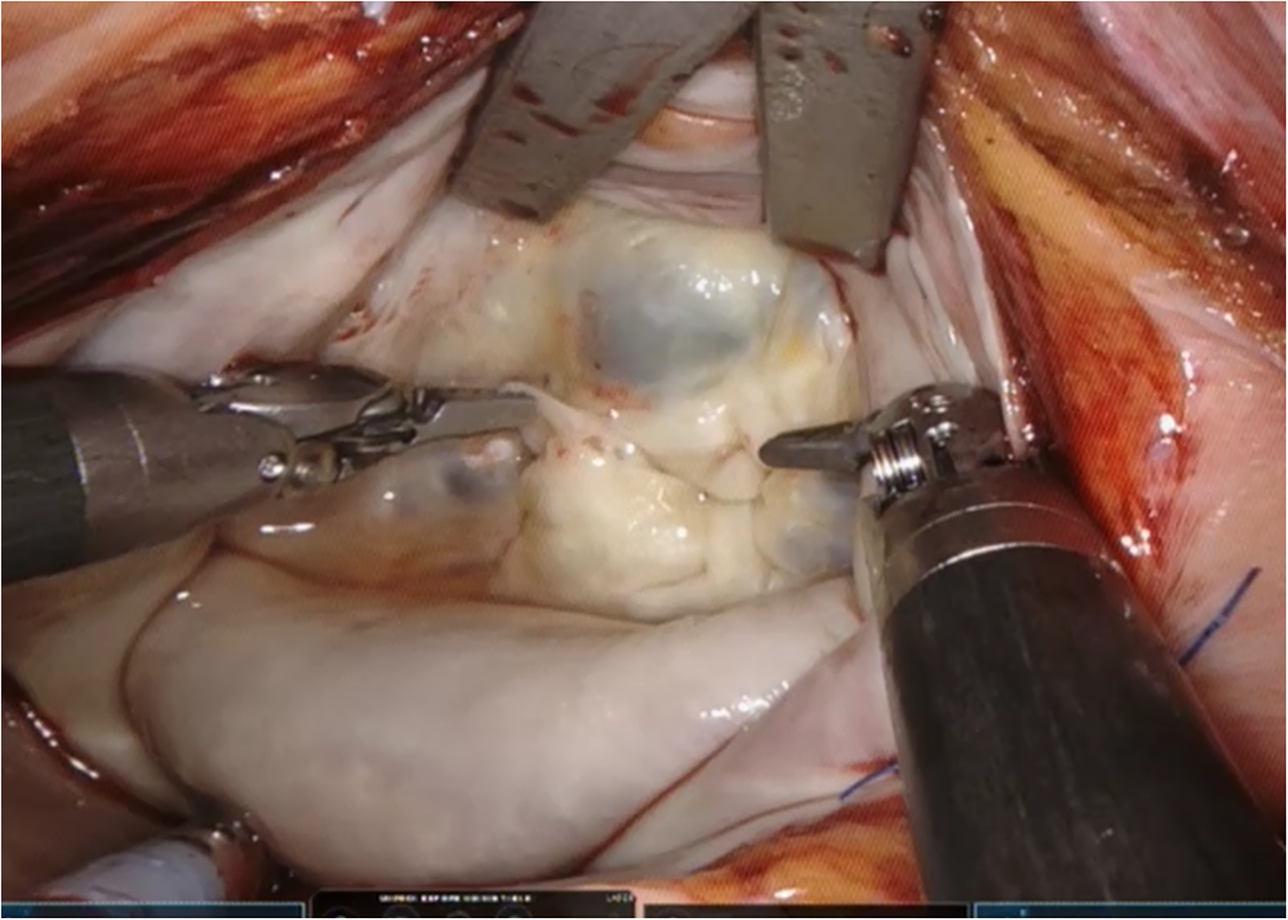
The operative view with the Da Vinci robot.

Three-dimensional video platforms (Einstein Vision® from BBraun (Melsungen, Germany) and Image1 S 3D from Storz (Tuttlingen, Germany)) have offset the loss of depth perception, but very few surgeons have mastered totally endoscopic surgery. Robotic surgery overcomes these issues and facilitates the adoption of totally endoscopic surgery. The Da Vinci robotic console (Intuitive Surgical, Sunnyvale, CA, USA) (Figure 1) allows immersion into the operative field through 3D-HD imaging, placing the surgeon inside the left atrium with a line of vision parallel to the flow of blood through the mitral valve (Figure 2). Finger and wrist movements are registered through sensors and translated into motion-scaled tremor-free movements avoiding both the fulcrum effect and the instrument shaft shear forces common to long-shafted endoscopic instruments. Wrist-like articulations at the ends of micro-instruments bring the pivoting action of the instrument to the plane of the operative field, improving dexterity in tight spaces and allowing for truly ambidextrous suture placement.

Robotic cardiac surgery has increased and plateaued in the US where approximately 1,700 robotic mitral valve procedures per year were performed from 2009 to 2015 (12). In Europe, robotic cardiac surgery is becoming more widespread, with a steep increase in the annual number of cases as new centres take up this pioneering technology (13, 14). However, recent changes in EU Medical Device Regulations affecting the Da Vinci robot are likely to temper this expansion until rectified.

One of the key challenges when introducing any less invasive technique has been the ability to demonstrate at least equal if not better results when compared to a gold standard of median sternotomy (15). Designing and conducting randomised controlled trials comparing operative techniques face several challenges. Blinding of patients and surgeons poses an obvious barrier, and it is also challenging to convince surgeons to randomise patients to a sternotomy in institutions with well-established robotic programmes and excellent outcomes. It is even more challenging to convince patients. This means that evidence pertaining to robotic mitral valve surgery is largely based on single centre experiences supplemented by large database analyses with propensity matching.

### Outcomes for robotic mitral valve surgery

#### Mortality

Operative mortality is consistently <1% in large series (16–18). Paul et al. found no difference between 631 propensity matched pairs of patients undergoing robotic-assisted and non-robotic mitral valve repair with respect to in-hospital mortality, complications, or composite outcomes in unadjusted or multivariable analyses (19).

#### Valve repair rates and durability

Similarly, valve repair rates and durability are equivalent to sternotomy. In fact, Hawkins et al. demonstrated a higher rate of repair in robotic procedures compared to sternotomy in a regional Society of Thoracic Surgeons (STS) database analysis from 2011 to 2016, despite similar rates of degenerative disease (20). This high rate of repair has been replicated in a large institutional series from the Cleveland Clinic which reported repair rates of 99.5% in almost 1,000 patients. Even in the learning curve, repair rates for degenerative pathology have been more than 98% (21, 22).

Experience reduces the rate of reoperation for repair failure from 7% in the first 100 cases down to 4.5% in the following 200 cases (23). Similarly, Murphy et al. also reported a fall from 6.8% down to 0.9% over 5 years in 1,257 patients (16). It was possible to redo the procedure robotically in 91% of cases. Five-year freedom from reoperations of 93.8% and 97.7%, and 5- and 6-year freedom from ≥2 + recurrent MR of 94.6% and 85% respectively provide evidence for repair durability (16, 24).

#### Operative durations

**Figure 3:**
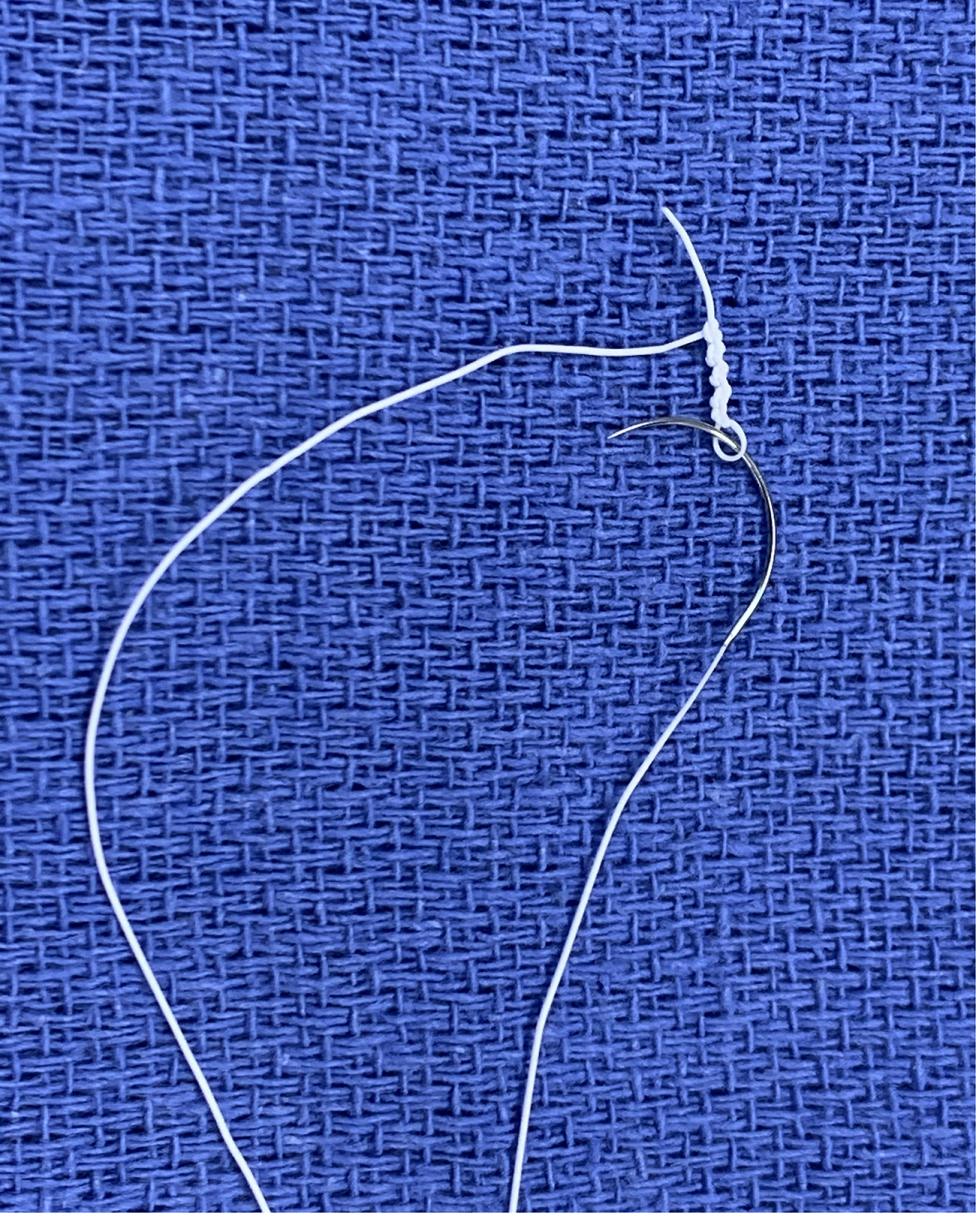
Pre-knotted suture made by knotting a CV4 Gore-Tex suture over a nerve hook.

These are longer than sternotomy, but decrease with increasing experience (25), which in turn is offset by increasing operative complexity as confidence with the technique develops (26). Facilitating techniques [continuous suture for annuloplasty band insertion (27) and pre-knotted sutures for left atrial closure (Figure 3)] and technologies (automated titanium clip knot fasteners, Cor-Knot®, LSI Solutions, NY, US) also serve to minimise this difference (28).

#### Morbidity

Many comparative series have demonstrated short-term advantages compared to sternotomy cases such as a reduced length of ICU and hospital stay, and faster return to normal activities, without a detriment to midterm outcomes (19, 20, 29, 30). This also translates into earlier return to paid employment on discharge (31). Data comparing robotic to video-assisted ‘*mini mitral’* surgery is lacking and conflicting, with the Cleveland Clinic showing advantages of robotic surgery (less AF and shorter hospital stay) (25) and the Virginia Cardiac Services Quality Initiative investigators showing disadvantages (more AF, more transfusions and longer hospital stay) (20).

An STS database analysis reported a 1.96 times higher rate of cerebrovascular accidents (CVA) in patients having less invasive mitral surgery, attributed to retrograde femoral perfusion (32). However, there were many confounding factors in this study including imprecise definitions of MIMVS, the effect of the substantial learning curve, retrospective comparisons of small historic cohorts with baseline differences and differing risk profiles for atherosclerosis, different methods of aortic occlusion and lack of reporting of peripheral vascular disease (PVD) or aortic assessment (33). Also, surgeon experience was likely a bias as the median number of less invasive mitral cases per centre per year was three. We have learnt from Prof Mohr's group in Leipzig that minimally invasive mitral surgery is an operation with a long learning curve (75–125 cases) with better results achieved in surgeons performing >1 case per week (34). In all other analyses, including in patients older than 65 years, no significant difference has been demonstrated between stroke rates in robotic and sternotomy approaches (25, 30). The largest randomized trial to date comparing mini-thoracotomy to sternotomy MV repair, the UK Mini Mitral trial, showed no difference in stroke rates, and this will hopefully now finally debunk this myth (35, 36).

The data for rates of blood transfusion and atrial fibrillation (AF) comparing robotic surgery to sternotomy is genuinely conflicting, with some reports suggesting a lower incidence of these (20), others showing no difference (22, 25) and others showing higher rates of these with robotic surgery than with the mini approach (30).

### Disadvantages

#### Costs and the learning curve

The cost associated with initiating a robotic cardiac service is substantial; investment in a surgical robot is followed by the ongoing financial commitment to device maintenance and purchase of reusable instruments. There is an increased burden on waiting lists as patient flow through the operating suite is reduced due to longer operative times. There is also a significant learning curve associated with adopting robotic cardiac surgery. Data from the Cleveland Clinic shows that the greatest reduction in operative times occurs during the first 200 cases with only modest reductions beyond this (17, 37).

There were equivalent costs between robotic and sternotomy MV repair in 631 propensity matched pairs of patients from the National Inpatient Sample from 2008 to 2012 (19). However, this data did not include the cost of the robotic system, maintenance, and amortization. Coyan et al. did, however, account for robotic capital depreciation and instrumentation costs and showed no significant differences in total costs of robotic mitral operations compared to a propensity matched group of sternotomy operations ($27,662 vs. $28,241, *p* = 0.27) (29). The initial higher capital investment associated with robotic surgery was balanced by the cost savings associated with reduced length of stay, reduced blood transfusions and reduced readmission rates when compared to the sternotomy group. We have demonstrated similar findings comparing video-assisted mini mitral to sternotomy MV repair (38).

Mihaljevic et al. compared the economic benefits of robotic mitral repair vs. alternative access via sternotomy, partial sternotomy and anterolateral thoracotomy taking into account purchase costs and maintenance as well as disposables (31). Income from return to paid employment as well as costs of postoperative care were also considered. Overall costs for robotic procedures were between 14% and 16% greater than alternatives but in centres performing 55–100 cases per year, this equilibrates over time with other techniques. In the majority of institutions, capital costs of the robot are spread across multiple surgical specialities and thus smaller volume robotic cardiac surgery programmes can still be economically viable (39). But is it unfair from an economic standpoint to consider the capital robot costs in a cost effectiveness analysis, as the cost of a hybrid room is not incorporated into analyses when comparing TAVR to surgical AVR?

Centre volume is key to the success of a robotic mitral valve repair programme not just for financial reasons but also ability to successfully navigate the learning curve. Lessons from experienced centres highlight good leadership and a dedicated team are vital and recommend a minimum number of 20 cases per year to maintain proficiency (40). Case selection is vital during the learning curve, and only high-volume centres have the caseload to do this in a temporally efficient manner. What is evident is that departments that have invested in their teams as much as their equipment see consistent uptake in robotic cases expanding to fill up to 90% of operative activity (16).

#### Lack of tactile feedback

In our experience, visual clues such as tissue deformation provide adequate information. Reiley et al. demonstrated that visual force feedback primarily benefits novice robot-assisted surgeons with diminishing benefits among experienced surgeons (41).

### Operative techniques

#### Patient preparation

We use the Da Vinci X (Intuitive Surgical) with four arms and 8 mm ports, and the thoracoscope in 30°-up orientation. We have TilePro enabled with feeds from the intra-operative transesophageal echo (TOE) and patient vitals. Bluetooth headsets facilitate clear communication between team members (Quail Digital, London, UK) (42).

Elevation of the right chest by 30° and placing a bolster sufficiently caudad under the right scapula to allow the humeral head to fall posteriorly, allows space for placement of the left arm port in the third interspace. Larger patients may require a long port.

Isolation of the right lung is needed, and this can be accomplished by either double lumen endotracheal intubation (DLETT), or a single lumen tube with a bronchial blocker. Each technique has its pros and cons, with our experience being that a DLETT gives more reliable lung isolation, especially when the right upper lobe bronchus has a high take-off, but with the disadvantage that it needs changing to a single lumen tube at the end of surgery.

For reoperations, we perform a ‘triple stick’ in the right internal jugular vein (IJV) to allow a balloon-tipped RV endocardial pacing wire to be floated across the tricuspid valve. This removes the need to dissect the diaphragmatic surface of the RV to place an epicardial temporary pacing wire. A pacing Swan-Ganz catheter is an alternative and can be used for primary operations also. If there is doubt that the diameter of the right internal jugular can accommodate a central venous catheter (CVC), 17Fr or 19Fr SVC drainage cannula and a transvenous pacing wire, the CVC can be placed in the left IJV.

#### Port positioning

The centre post of the patient cart should be level with the camera port. If the post is too caudal then arms 3 and 4 lie too flat and will conflict externally. Usually, the fourth intercostal space is used for both the access port and camera port. If the patient has a short thoracic cavity, then the third interspace may be used. We have found that a reliable indicator of the correct interspace is the one overlying the right inferior pulmonary vein on the chest x-ray. The right arm port is placed two interspaces caudal to the access port in the anterior axillary line. To avoid conflict of the right elbow with the left iliac crest, the table is rolled to the left. The left atrial retractor port goes in the 4th or 5th interspace, medial to the midclavicular line.

#### Cannulation

Placement of an internal jugular drainage cannula (17 or 19Fr Biomedicus, Medtronic, Dublin, Ireland) in addition to a femoral venous cannula ensures adequate venous drainage in all patients. This is of paramount importance as poor venous drainage will compromise right heart protection. Advancing the IVC cannula into the SVC allows improved mitral exposure as the cannula acts like a rod in the RA, allowing the LA retractor to lift against it. It is therefore advantageous to use a stiffer venous cannula (e.g., Biomedicus, Medtronic) as opposed to a more flexible one (e.g., 23/25Fr RAP femoral venous dual stage, LivaNova, UK).

#### Cardiopulmonary bypass and myocardial protection

As previously stated, operative times are prolonged during the initial learning curve, therefore we advocate systemic cooling to 28–30°C which confers the benefit of both improved myocardial protection and protection of the right lung to avoid unilateral pulmonary edema (43). Unilateral pulmonary edema occurs in <1% of cases but it is associated with a 33% mortality. This is likely to be a result of ischaemia-reperfusion injury of the right lung. Preventative measures include minimising the duration of single lung ventilation, maintenance of systemic pressure on cardiopulmonary bypass (CPB) ≥ 65 mmHg, maintaining haematocrit, and active cooling.

We advocate the use of single dose cardioplegia (e.g., Custodiol® or del Nido) for myocardial protection. It is helpful to avoid redosing, especially if using the IntraClude device. Crystalloid cardioplegia has the benefit of lower line pressures during infusion when compared to blood cardioplegia and is therefore particularly useful when using the IntraClude device. Beware, however, of repeated saline testing washing out the cardioplegia and compromising myocardial protection.

#### Intraclude or chitwood transthoracic clamp

There are, broadly speaking, two techniques for robotic mitral valve surgery based on the technique of aortic occlusion and both are highly reproducible.

**Figure 4:**
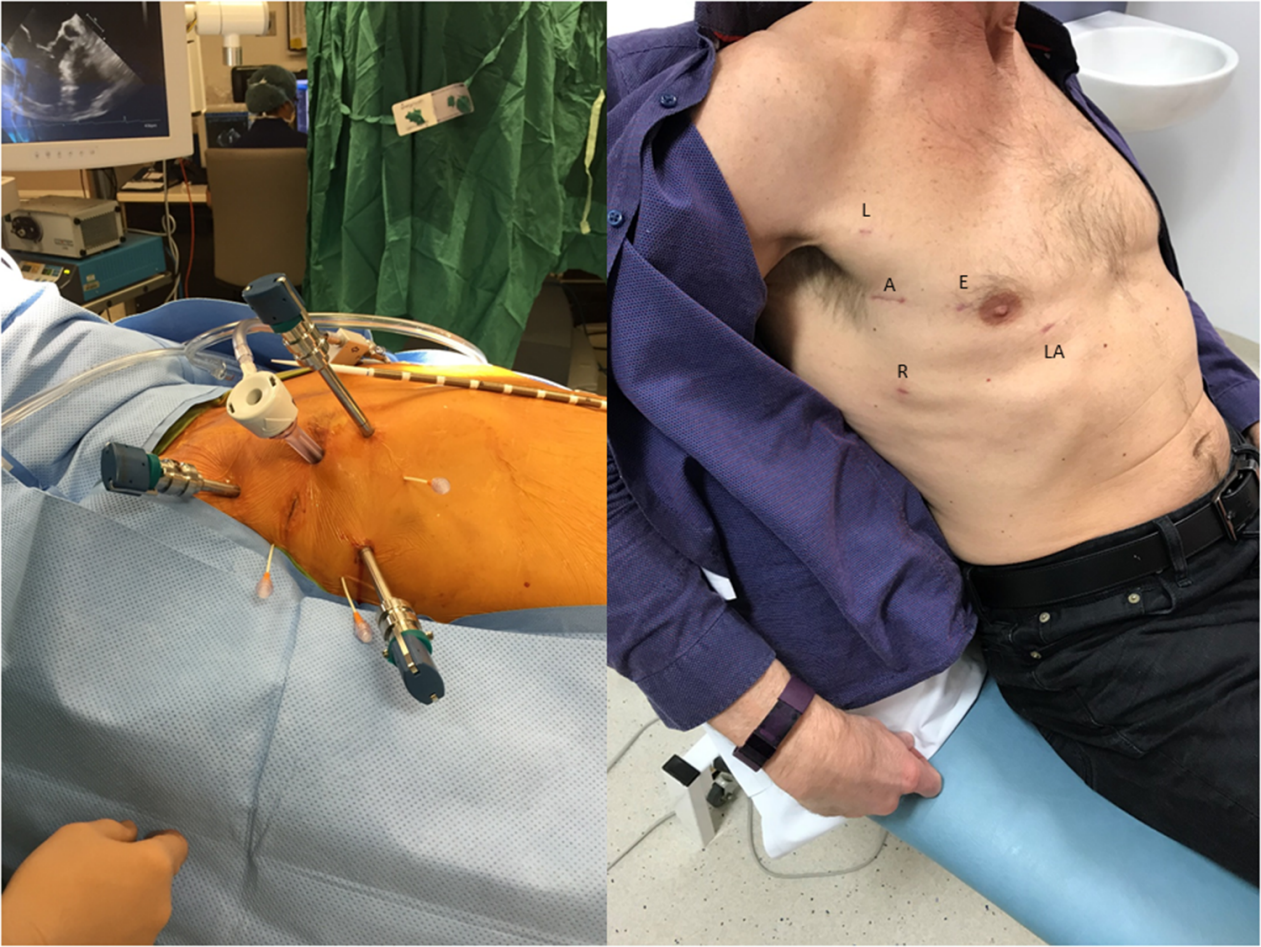
The LEAR technique (Lateral Endoscopic Approach for Robotics). A, access port; E, endoscope port; L, left arm port; LA, left atrial retractor port; R, right arm port.

1. **The LEAR Technique** (Lateral Endoscopic Approach for Robotics) was devised by Dr Douglas Murphy (Atlanta, USA) and is a port-based totally endoscopic approach using four 8 mm ports and one 20 mm flexible access port (16) (Figure 4). With a port-based approach, an endoaortic balloon is used to occlude the aorta (IntraClude, Edwards Lifesciences, Irvine, CA) because cannulating the aortic root can be challenging. This is the least invasive option but introduces complexity in the positioning and management of the IntraClude to ensure adequate aortic occlusion and myocardial protection are maintained. Maintaining the position of the endoballoon needs constant vigilance and is affected by the pressure in the aorta and the tension on the catheter. Therefore, bilateral radial arterial lines are required to detect distal migration which can occlude the origin of the innominate artery (e.g., if the systemic blood pressure falls).

Due to the IntraClude being deployed through a side arm on the femoral arterial cannula, it can lead to high arterial line pressures. It may therefore be necessary to cannulate both femoral arteries to achieve optimal flow and line pressure on CPB.

**Figure 5:**
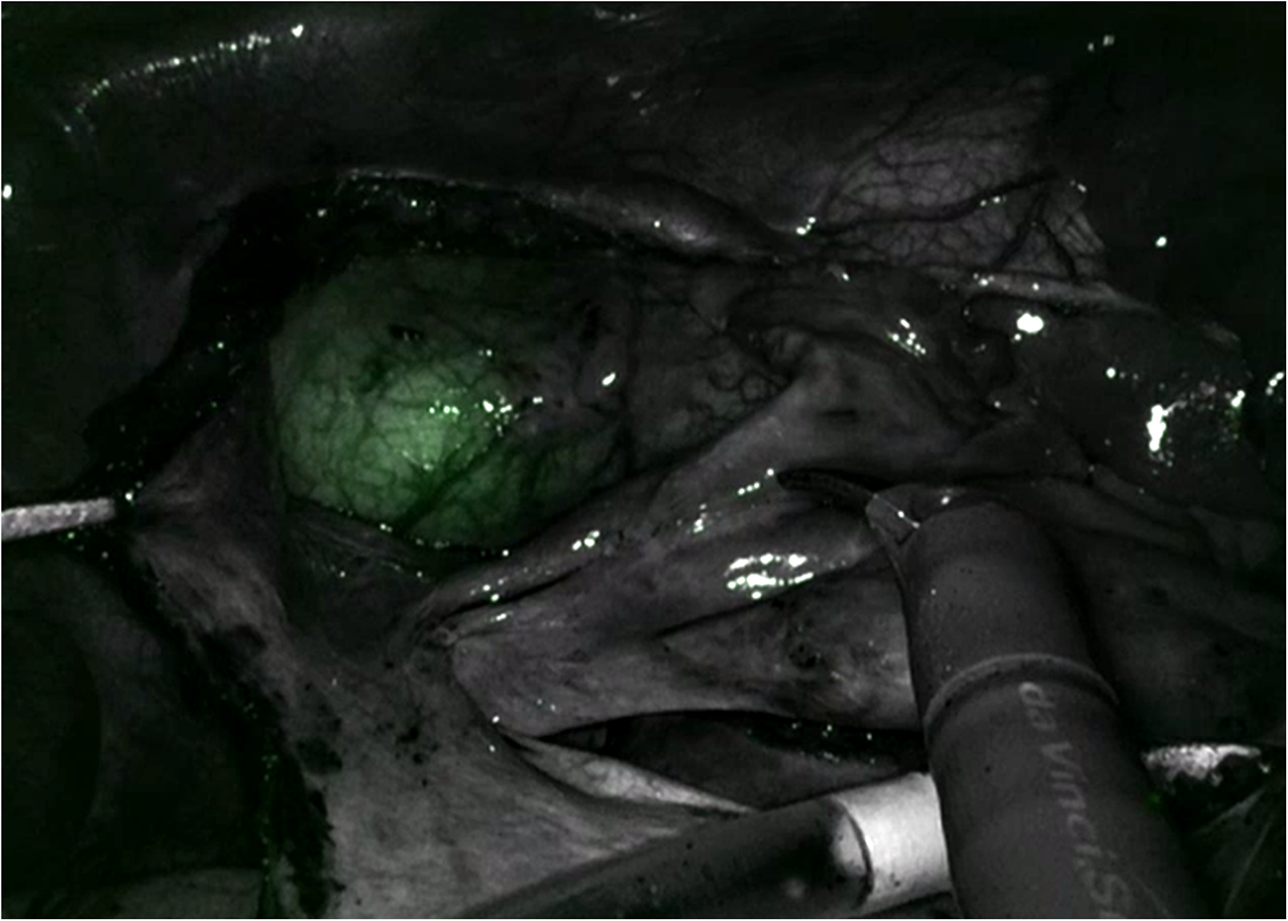
Indocyanine green fluorescing in the intraClude in the mid ascending aorta.

One innovative development to assist with Intraclude placement is the use of an albumin and indocyanine green (ICG) solution to fill the balloon (Figure 5). This allows image-guided placement of the IntraClude device, as the solution fluoresces with the Firefly fluorescence imaging of the Da Vinci system (44). The disadvantages of the IntraClude include that it is single use and expensive (GBP £1,500). In a healthcare system with constrained resources this cost is not negligible, especially when this is added to the additional cost of the robotic procedure. For instance, each of the five instruments has a limit of 10 uses and at £200 per use, this equates to an additional £1,000 per case.

2. **The Chitwood Technique**—this was popularised by Dr Randolph Chitwood (Greenville, NC) using a 4 cm minithoracotomy, through which the 3D scope is placed, and three 8 mm ports. The eponymous Chitwood clamp is placed transthoracically to occlude the aorta. Unlike the IntraClude, this has the advantage that it does not migrate, it provides reliable aortic occlusion and is reusable and thus more cost effective. As no additional catheters are placed via the femoral arterial cannula, it is rarely necessary to perform bilateral femoral cannulation. This provides a significant cost saving and importantly makes the overall procedure less complex but does have some disadvantages. Firstly, a minithoracotomy is needed rather than a truly port-based approach and the operation is therefore similar to a video-assisted ‘mini mitral’; secondly, there is the potential for conflict between the clamp and the left robotic arm but this is usually easily managed; thirdly, it necessitates a short 2nd bypass run to decannulate and control the root of the aorta, which can occasionally lead to troublesome bleeding.

A recent development has been the development of a port-only endoscopic technique using the Chitwood clamp (45). This is likely to prove useful in patients with aorto-iliac pathology or limited femoral artery access, or robotic teams who employ transthoracic aortic clamping through a thoracotomy and want to facilitate the transition to a port-only robotic approach.

#### Mitral valve repair techniques and annuloplasty band

Standardised mitral valve repair techniques can be utilised with the robotic platform but our preference has been to translate the Gore-Tex loop technique we use during video-assisted ‘mini mitral’ surgery to the totally endoscopic robotic environment. Gore-Tex loops are widely used in Europe and have proven durability, but their use in robotic surgery has not been widely reported. We have previously reported a modified technique using a short length of CV4 Gore-Tex suture to measure the loop length and demonstrated how to adjust the ‘effective length’ of fixed length loops if the initial length measurement is suboptimal in the completed repair (46). The loop technique simplifies the repair of complex multi-segment prolapse, and its wider adoption during robotic surgery will allow more patients with increasingly complex valves to benefit from totally endoscopic reconstruction. Recent reports have demonstrated that experienced robotic cardiac programmes can offer the benefits of robotic surgery to those patients who are at high risk or require complex mitral valve reconstruction (47–49).

Most surgeons prefer to use flexible bands due to ease of implantation with the robotic system, but semi-rigid bands and rings can also be implanted depending on preference. Interrupted 2/0 braided polyester secured with Corknot (LSI solutions, NY, USA) or continuous sutures with either CV4 or 2/0 braided polyester can be used to secure the band (27).

A motorised saline insufflator (e.g., StrykeFlow, Stryker, MI, USA) is used to test the valve repair whilst monitoring the root pressure on TilePro. Suction should be maintained on the root vent until air has been displaced from the aortic root. We aim for a symmetrical closure line at least 70% posteriorly and with less than 9–10 mm of A2 coaptation on ink testing—any more than this invariably predicts the risk of systolic anterior motion (SAM). Any imperfections at this stage are easy to correct as the loop is simply detached and either lengthened, shortened, or repositioned. This is one of the advantages of the loop technique compared to individual neochords which cannot be adjusted once tied and must be explanted.

## Discussion

With one hundred years of experience operating on the mitral valve, cardiac surgeons, cardiologists, and cardiac anaesthesiologists have come a long way in developing a close collaborative team-based approach to mitral valve pathology. Surgery has clear advantages in resolving primary degenerative MR and restoring life expectancy compared to that of an age and sex-matched population. Excellent pre-operative echocardiographic assessment of the valve allows the surgeon to plan the repair strategy and predict repair success. Surgeons are now able to offer surgical correction of MR through port-based totally endoscopic robotic approaches thus allowing rapid return to normal activities in a few weeks rather than a few months with a sternotomy. The future lies in a multi-disciplinary approach with decision making shared with the patient where open, minimal access, totally endoscopic robotic and transcatheter techniques are considered for each patient. The number of robotic cardiac procedures being performed globally is expected to continue to rise as experience grows with robotic surgical techniques and increasing numbers of cardiac surgeons become proficient with this innovative technology. This will be facilitated by the introduction of newer robotic systems and increasing patient demand. Well-informed patients will increasingly seek out the opportunity of the least invasive surgery in reference centres in the hands of a few highly experienced robotic surgeons.
